# Physical Activity Trends in Korean Adults from Korea National Health and Nutritional Examination Survey from 2014 to 2019

**DOI:** 10.3390/ijerph19095213

**Published:** 2022-04-25

**Authors:** Hoyong Sung, Geonhui Kim, Xiaonan Ma, Harim Choe, Yunmin Han, Jiyeon Yoon, Yeun Ryu, Yeon Soo Kim

**Affiliations:** Department of Physical Education, Seoul National University, Seoul 08826, Korea; hoyongsung86@snu.ac.kr (H.S.); masazang@snu.ac.kr (G.K.); adamas0505@snu.ac.kr (X.M.); dw031301@snu.ac.kr (H.C.); hym224@snu.ac.kr (Y.H.); dwldyd@snu.ac.kr (J.Y.); yeunryu@snu.ac.kr (Y.R.)

**Keywords:** physical activity, Korea National Health and Nutritional Examination Survey

## Abstract

The current study aimed to examine the adherence trend for aerobic and muscle-strengthening physical activity (PA) guidelines among Korean adults using the Korea National Health and Nutritional Examination Survey from 2014 to 2019. Korean adults (*N* = 2642) were included in the current study to examine the trend of PA adherence from 2014 to 2019. The PA guidelines are: (a) aerobic activity (a minimum of 150 min moderate to vigorous PA weekly) and (b) muscle-strengthening activity (a minimum of two days weekly). Self-report questionnaires in the survey measured both activities. The adherence to PA guidelines by survey year was examined using a multivariable logistic regression analysis adjusted for covariates. There was a significant decreasing trend in which the adherence rate to aerobic PA guidelines changed from 57.0% in 2014 to 45.6% in 2019 (*p* < 0.001). On the other hand, the trend in adherence rate to muscle-strengthening activity was not significant (*p* = 0.976). The adherence rate to muscle-strengthening activity guideline was slightly increased but still low from 20.8% to 23.3% during 2014–2019. The aerobic PA guidelines are significantly decreasing, and more than half of the Korean adults in 2019 have not followed the guidelines. In addition, there has been a slight increase in muscle-strengthening activity; however, the adherence rate is meager. Therefore, this study suggests that Korean adults’ aerobic and muscle-strengthening PA participation is necessary for their low adherence rate and decline tendency.

## 1. Introduction

Evidence for the health-related benefits of physical activity (PA) has been systematically assessed and evaluated elsewhere [1]. In addition, globally, evidence-based PA guidelines have been adopted to evaluate a person’s PA behavioral risk for health [1,2,3].

For adults, WHO PA guidelines are at least 150 min of moderate to vigorous aerobic PA per week and at least two days of muscle-strengthening activity per week, respectively—depending on the type of activity [4].

Few people in the U.S. and U.K. were knowledgeable of the aerobic PA guidelines [5,6,7]. In the U.S., only 0.56% of adults were familiar with the moderate to vigorous aerobic PA guidelines [6]. The proportion of U.K. adults who recalled the guidelines was 18% in 2013 and 11% in 2007 [5]. On the other hand, 25% of Finnish young adults correctly identified the muscle-strengthening activity guideline [7]. It is less known how many people know about the muscle-strengthening activity guideline since there is a scarcity of papers published.

Based on guidelines adherence, the global trend for the prevalence of insufficient aerobic PA was stable from 28.5% in 2001 to 27.5% in 2016 [8]. However, the prevalence trend of the muscle-strengthening activity guideline has not been studied much yet compared to aerobic PA. For example, a recently published study stated that U.S. adults only followed the muscle-strengthening activity guideline from 29.1% in 2011 to 30.3% in 2017 [9].

From a prospective cohort study, people meeting both aerobic and muscle-strengthening activity guidelines had lower all-cause mortality compared to meeting only one guideline [10]. Moreover, a few recent cross-sectional studies showed results that suggested that meeting both guidelines was associated with a lower risk of having adverse cardiometabolic biomarkers [11], chronic health conditions [12], and obesity [13].

The prevalence of meeting both guidelines has been about half of the prevalence of meeting only the aerobic PA guidelines since 1997 in the U.S. [14]. Historically, PA recommendations have been considered as only comprising of aerobic PA, which was mainly highlighted until muscle-strengthening activity recommendation was presented [12]. Therefore, surveillance of the prevalence of meeting both guidelines would be needed according to the importance related to health.

To our knowledge, few studies have been published about the prevalence of meeting both guidelines from other countries than Western countries like the U.S. [11]. Therefore, the current study aimed to examine the adherence trend for both the aerobic and muscle-strengthening activity guidelines among representative Korean adults.

## 2. Materials and Methods

### 2.1. Participants

The current study used the Korea National Health and Nutritional Examination Survey (KNHANES) 2014–2019. The Korea Center for Disease Control administers KNHANES and an ongoing—since 1998—periodic cross-sectional survey for gathering information about health behavior, the prevalence of chronic and other diseases, and food and nutritional intake of the non-institutionalized Korean population through a stratified, multi-stage probability sampling design. The sampling design reflects the primary sample units, strata, and weight for KNHANES, which has been applied to acquire unbiased estimates of the Korean population. In addition, trained professionals conducted health-related surveys and physical examinations in the mobile examination center, and a complete description of the survey process is available elsewhere [15].

Participants aged over 19 years were initially included (*N* = 33,561) and excluded if participants had missing or invalid data on the PA data from the current study. The final 27,642 participants were included in the current study.

The data before KNHANES 2014 was not considered for the current study since the questionnaire for the PA was not the same as the one used during the KNHANES 2014–2019. The Research Ethics Review Board has approved KNHANES of the Korea Centers for Disease Control and Prevention, and all the participants provided written informed consent (IRB No. 2013-12EXP-03-5C, 2018-01-03-P-A). The current study was exempted by the Seoul National University Institutional Review Board since we used de-identified data (IRB No. E2202/004-005). This study followed the Strengthening the Reporting of Observational Studies in Epidemiology (STROBE) reporting guideline [16].

### 2.2. Data Collection

All raw data were downloaded from the KNHANES website. PA was measured by self-reporting using the Global PA Questionnaire (GPAQ), which was developed by the World Health Organization (WHO) [17,18]. The GPAQ was translated and validated over the country [19]. The GPAQ asks for at least 10 min of continuous PA over three domains (i.e., work, transportation, and leisure) during a typical week, and, more specifically, asks about the intensity (i.e., moderate and vigorous), frequency (i.e., days a week), and duration (i.e., hours and minutes a week) of PA. Moderate-intensity PA was defined as 4.0 metabolic equivalent (MET) PA that required moderate physical effort that caused small increases in breathing or heart rate [18]. Vigorous-intensity PA was defined as 8.0 MET PA that required hard physical effort that caused significant increases in breathing or heart rate [18]. Work- and leisure-related PA domains included both moderate and vigorous-intensity PA questions, but not for the other transportation-related ones since it was defined as only moderate-intensity if it was activities such as walking or cycling. The total minutes of PA was calculated as minutes per week for moderate to vigorous PA (MVPA) to examine the adherence to the PA guidelines. The MVPA total minutes per week was calculated as moderate-intensity plus twice the minutes of vigorous-intensity PA from all work-, leisure-, and transportation-related PA domains. Participants were classified as adhering to the aerobic PA guidelines if they had at least 150 min per week of MVPA. The PA data were scrutinized for whether they were measured as valid or not using the WHO analysis guide [18].

The muscle-strengthening activity was defined as the weekly frequency of muscle-strengthening activity. Participants self-reported the following question: “Over the recent one week, how many days did you do muscle-strengthening activities such as push-up, sit-up, and using a dumbbell, bar-bell, and pull-up bar”. Participants were classified as adhering to the muscle-strengthening activity guideline if they had at least two days per week of this activity.

The covariates, such as age, sex, body mass index (BMI), family income, educational level, smoking status, and alcohol intake, were included to examine their associations with PA guidelines. Covariates were chosen due to their association with PA and comparison with previously published studies [9,11,12,14,20,21,22,23].

Age was categorized into six groups (i.e., 19–29, 30–39, 40–49, 50–59, 60–69, ≥70). The trained technician measured height and weight. BMI was calculated as weight (kg) divided by height (m) squared. BMI was grouped as underweight (<18.5), normal (18.5–24.9), overweight (25–29.9), and obese (≥30) by following WHO guidelines. Family income was expressed through a quartile range (i.e., low, mid-low, mid-high, high). Educational level was categorized into three groups (i.e., less than high-school, high-school graduate, and more than college or above). Smoking status was categorized as a non-smoker, past smoker, and current smoker who had smoked at least five packs of cigarettes. Alcohol intake was categorized as non-drinking, moderate drinking (drink at most 2–4 times a month), and heavy drinking (drink at least 2–3 times a week and at least seven glasses for males and five glasses for females a day).

## 3. Statistical Analyses

All analyses were conducted using the SVYSET module of STATA version SE 16.1 (StataCorp., College Station, TX, USA) to account for the stratified, multi-stage probability sampling design. A two-sided *p*-value of <0.05 was considered to be statistically significant.

The characteristics of the participants were described as unweighted numbers and weighted percentages for the categorical variables. Chi-squared tests were used for distributional differences in the 2014 to 2019 survey year characteristics. The weighted adherence rate and 95% confidence interval (CI) for each stratum in characteristic variables across survey years were described. A multivariable logistic regression that was adjusted for the covariates was used to examine the adherence trends to the aerobic and muscle-strengthening activity guidelines, as well as when they were combined.

The independent and dependent variables for the analysis were survey year and binary adherence response. In addition, the covariates mentioned above, such as age, BMI, family income, educational level, smoking status, and alcohol intake, were included to eliminate possible confounders in the relationship between the survey year and adherence response.

In the multivariable logistic regression analysis, *p*-values for linear trends across the survey year and interaction between the strata of characteristic variables and the survey year were calculated. The *p*-values for the linear trends in adherence rate were calculated after including the survey year as a continuous term in the multivariable logistic regression analysis. In addition, the interaction analyses were conducted to examine whether the trends in adherence rate differ across the strata of each characteristic variable.

## 4. Results

The distribution of the population over years was significantly different in BMI, educational level, smoking, and alcohol intake (Table 1).

There was a significant decreasing trend in which the adherence rate of aerobic PA changed from 57% in 2014 to 45.6% in 2019, as presented in Table 2 (*p* < 0.001 for trend). The decreasing trend was significant in the subgroup of all variables except for those who were underweight. The comparable difference in aerobic PA guideline adherence between 2014 and 2019 was presented in 50–59 years (15%) and the high school graduate (14%) groups. In addition, the less aerobic PA guideline adherence rate was presented relatively in the 70 years or above, female, underweight, low income, lower than high school graduate, non-smoking, and non-drinking groups, respectively.

On the other hand, the trend for the adherence rate to muscle-strengthening activity guideline was not significant (Table 3, *p* = 0.976 for trend). However, there was a significantly increasing trend only in the 30–39 years and the group for high family income. The decreasing muscle-strengthening PA guideline adherence between 2014 and 2019 was presented only in the obesity and non-drinking groups. In addition, the lower muscle-strengthening PA guideline adherence rate was presented relatively in the 70 years or above, female, obese, low income, lower than high school graduate, non-smoking, and non-drinking groups, respectively.

The adherence rate trend for combined aerobic and muscle-strengthening activity guidelines was not significant (Table 4, *p* = 0.051 for trend). However, there was a significantly decreasing trend in the 40–59 years, the mid-low family income, and the less than high school graduate group.

**Figure 1:**
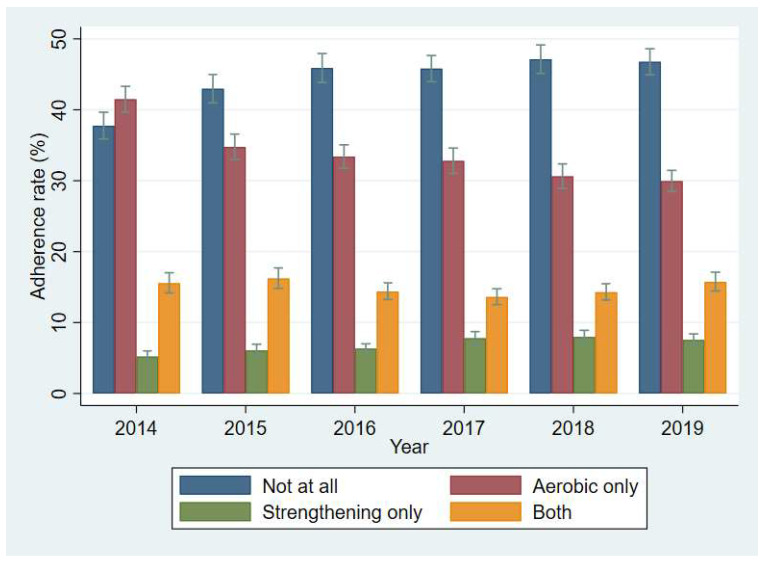
Trends of the adherence rate to the PA guidelines by survey year.

People who did not meet the guidelines for combined both aerobic and muscle-strengthening activity had a significant increasing trend (Figure 1. *p* < 0.001).

## 5. Discussion

During 2014–2019, the aerobic PA guidelines adherence rate for Koran adults decreased significantly. Although the adherence rate of the muscle-strengthening activity guideline slightly increased, but not significantly, from 20.8% to 23.3%, it still showed a low adherence rate.

Previous studies investigating the trends in aerobic PA in Korean adults before 2014 also reported a gradual decrease [24,25]. Kang et al. (2015) [24] examined the trends in aerobic PA in Korean adults using a walking rate defined as walking for at least 30 min a day over five days per week. In this study, the aerobic PA participation was 50.6% in 2008, but it decreased to 37.5% in 2014. In addition, by using the international PA questionnaire, Kim (2017) [25] reported that Korean adults’ aerobic PA participation rate dropped from 41% in 2008 to 30.8% in 2012 and 33.6% in 2013 through analyzing the KNHANES data, which was similar to our study. Although there are differences in the definition and measurement tools of aerobic PA, these findings are consistent with our results, which indicated that the adherence rate in Korean adults to aerobic PA guidelines had gradually decreased. As opposed to aerobic PA, in the current study, the muscle-strengthening activity participation rate did not show a significant trend with a change in adherence from 20.8 to 23.3% from 2014 to 2019. A previous study examining Korean adults’ muscle-strengthening activity participation rates from 2008 to 2013 showed a constant trend from 20.9 to 22.5% [25].

Accordingly, in Korean adults, aerobic PA has been shown to likely decrease continuously, and the muscle-strengthening activity participation rate has maintained a constant level. The significant decreasing trend of the aerobic PA adherence rate might be related to people’s increasing overall adiposity levels as lifestyles have changed over time [26]. Furthermore, as sedentary lifestyles become more common, since the proportion of screen-based tasks and numbers of sedentary jobs increased, aerobic capacity could have been changed, and this phenomenon may contribute to a significant decrease in PA level [27]. A high amount of sedentary time, an emerging topic among contemporary humankind, is also becoming a rising issue in Korea, having the second-longest number of working hours in OECD countries [28]. A study examining the changes in PA during the lifecycle reported that people’s lifestyle has gradually shifted from sedentary to moderately active or active as they reach reproductive age and return to sedentary again after the age of 50 years [29], which may explain the phenomenon of Koreans in their 50s showing a significant decrease in the rate (5%) of PA level compared to other age groups.

The difference in the muscle-strengthening activity adherence rate between males and females is consistent with the results of previous studies [12,29,30,31], finding that men have higher adherence rates than women. However, in terms of adherence trends for muscle-strengthening activity, there was a significantly increasing trend only in the 30–39 years old and the high family income group from 2014 to 2019. For those who do not know bodyweight exercises, muscle-strengthening equipment will be easier to use to exercise. Governments might help provide several spaces where this equipment is installed. In addition, it is known from previous studies that bodyweight exercise has the same health benefits as gym-based strengthening activities [10]. Therefore, it is crucial to change people’s awareness around the difficulty of participating in muscle-strengthening activities.

Both aerobic and muscle-strengthening activity adherence was recommended for various health benefits. However, in the current study, the proportion of people who did not adhere to both aerobic and muscle-strengthening activity guidelines has increased significantly (Figure 1. *p* < 0.001). Although there was a lack of evidence, the decreased proportion of people who did not meet both aerobic and muscle-strengthening activity guidelines may be contributing to the decreasing trend in meeting aerobic PA guidelines alone. The current study’s findings differed from previous studies [32,33,34,35] that examined the trend of aerobic and muscle-strengthening activity. For example, a study using U.S. adults over 18 suggested that the prevalence of not meeting both aerobic and muscle-strengthening activity significantly decreased from 56.6% to 42.3% from 1998 to 2018 [30].

Moreover, the trend of meeting guidelines for both aerobic and muscle-strengthening activity was seen as insignificantly unchanged in this study. Several studies using data from English adults [31,32] similarly showed that the proportion of adherence to both PA guidelines was unchanged in men and women between 2012 and 2016 (men, 31.0% and 30.0%; women, 22.0% and 23.0%, respectively). On the contrary, the studies examining the trend of meeting PA guidelines in the U.S. and Dutch population reported that both aerobic and muscle-strengthening activity guidelines adherence increased over time (14.4% to 24.0%; 39.9% to 46.0%, respectively) [30,33].

A lack of aerobic PA is associated with chronic diseases such as the risk of CVD, type 2 diabetes, high blood pressure [34], quality of life [35], all-cause mortality [36], and medical expenditure [37]. For people who participate in aerobic PA for more than 150 min a week, all-cause mortality and CVD risk are reduced by up to 75% [38]. However, the current study found that the aerobic PA adherence rates decreased from 57% in 2014 to 45.6% in 2019 in Korean adults. The most recent global estimates showed that one in four adults (27.5%) [8] and more than three-quarters (81%) [39] of adolescents do not meet the recommended amount of aerobic exercise. However, according to Ekblom et al., (2010) [40], people with high cardiovascular fitness and low PA showed a high risk of CVD, though people with high PA showed a relatively low risk even if their cardiovascular fitness was low [40]. Therefore, just participating in PA itself might provide various health benefits.

PA guidelines need to be encouraged through various sources, especially through government efforts, which are necessary within the national and social dimensions. In addition, national policies or goals to promote PA should also be well established [4]. The current study showed that Korean adults increased their muscle-strengthening activity adherence slightly, but the proportion of adults who met the muscle-strengthening activity guidelines was half that of the adherence rate of the aerobic PA guidelines. Only one in five Korean adults adhered to weekly muscle-strengthening activity guidelines. Muscle-strengthening activity is closely related to various diseases. Briefly, muscle-strengthening activity reduced the risk of diabetes [41], enhanced cardiometabolic and musculoskeletal health [42], improved mental health [43], and decreased all-cause mortality [10]. However, despite this evidence, muscle-strengthening activity has been pushed back from priorities in public health compared to aerobic PA [44,45]. There is a lack of effort in promoting muscle-strengthening activity guideline and policies; thus, people are less aware of the importance of muscle-strengthening activity [45,46]. The government needs to provide campaigns supporting the importance of muscle-strengthening activity for individual health, attractive spaces for muscle-strengthening activity, and increase equipment availability to encourage muscle-strengthening activity [46].

As mentioned above, it is well established that participation in aerobic and muscle-strengthening activity has several health benefits, respectively. Furthermore, previous studies have investigated the effects of meeting both guidelines for aerobic and muscle-strengthening activity. In this regard, several studies demonstrated that people who meet both aerobic and muscle-strengthening activity guidelines were associated with the lowest prevalence of obesity, depressive symptoms, and sleep disorders [13,47,48].

In addition, the other study examining 383,928 US adults suggested that meeting both guidelines had the lowest prevalence ratios for adverse cardiovascular conditions, such as hypertension, diabetes, myocardial infarction, coronary heart disease, and stroke [12]. Moreover, Dankel et al. (2017) [49] analyzed data contained with accelerometer assessed PA, a more objective method for aerobic PA. The results indicated that meeting both guidelines had the lowest odds ratios for multi-morbidity and metabolic syndrome than those who met neither [49,50]. Although these were beneficial effects for health, the adherence rates for meeting both guidelines in Korean adults were unchanged over time. Therefore, strategies and efforts such as providing advertisements and local community programs to promote aerobic and muscle-strengthening activity guidelines are needed to increase adherence to PA guidelines.

This study has several strengths. First, the current study used representative and reliable data for Korean adults through KNHANES. In addition, the latest PA trend study uses the latest public data. Second, the independent trends by year and independent trends according to the subgroups of the covariates have been examined and adjusted for the covariates. Third, the adherence trends for the muscle-strengthening activity guidelines and the combined guidelines for both aerobic and muscle-strengthening activity were examined in the current study, which has been scarcely studied. However, the year from 2014 to 2019 was a relatively short-term to examine the trends in PA. Second, aerobic PA was estimated by the questionnaires, which is usually overestimated compared to people’s valid aerobic PA.

## 6. Conclusions

The current study raises awareness of decreasing trend of aerobic PA and low muscle-strengthening activity in Korean adults. Therefore, future national efforts to enhance people’s physical activity for both aerobic and muscle-strengthening must be emphasized.

## Figures and Tables

**Table 1:** Characteristics of participants by survey year.

	Number (%) of Participants		Unweighted Number of Participants (Weighted %) by Survey Year						
	Unweighted *N* (%)	Weighted *N* (%)	2014	2015	2016	2017	2018	2019	*p*-Value
Total Sample Size	33,561	232,793,663	4981	5075	5807	5816	5963	5919	
Age (year)									
19–29	3346 (12.1)	7,080,854 (18.4)	581 (19.2%)	645 (19.0%)	668 (17.8%)	704 (18.1%)	748 (18.2%)	723 (17.8%)	0.795
30–39	4332 (15.7)	7,033,244 (18.3)	862 (19.4%)	696 (18.4%)	1036 (18.4%)	857 (17.9%)	881 (17.6%)	882 (17.2%)	
40–49	4985 (17.9)	7,794,451 (20.3)	850 (20.5%)	884 (20.3%)	1081 (20.7%)	1062 (20.2%)	1081 (19.8%)	1076 (19.6%)	
50–59	5351 (19.4)	7,602,818 (19.8)	959 (19.8%)	1052 (19.7%)	1052 (19.9%)	1148 (20.0%)	1140 (19.5%)	1131 (20.1%)	
60–69	4908 (17.8)	4,812,759 (12.5)	892 (11.6%)	939 (12.1%)	972 (12.5%)	1039 (13.0%)	1066 (13.4%)	1071 (14.1%)	
70 or above	4747 (17.2)	4,117,220 (10.7)	837 (9.6%)	859 (10.5%)	998 (10.8%)	1006 (11.0%)	1047 (11.6%)	1036 (11.3%)	
Sex									
Male	11,910 (43.1)	18,920,993 (49.2)	2071 (48.9%)	2179 (48.6%)	2487 (49.4%)	2572 (49.7%)	2601 (49.6%)	2617 (49.6)	0.823
Female	15,732 (56.9)	19,520,353 (50.8)	2910 (51.1%)	2896 (51.4%)	3320 (50.7%)	3244 (50.3%)	3362 (50.4%)	3302 (50.5)	
Body Mass Index									
Underweight	1080 (3.9)	1,629,190 (4.2)	227 (4.9%)	200 (4.6%)	218 (4.0%)	226 (4.1%)	209 (3.7%)	235 (4.3%)	0.004
Normal	17,080 (61.8)	23,658,858 (61.6)	3197 (64.1%)	3127 (61.7%)	3544 (60.5%)	3562 (61.1%)	3650 (60.6%)	3651 (61.1%)	
Overweight	7982 (28.9)	11,019,923 (28.7)	1341 (26.7%)	1482 (28.4%)	1723 (29.8%)	1706 (29.1%)	1730 (29.2%)	1684 (28.5%)	
Obesity	1500 (5.4)	2,133,375 (5.6)	216 (4.4%)	266 (5.3%)	322 (5.7%)	322 (5.7%)	374 (6.5%)	349 (6.2%)	
Family Income									
Low	5277 (19.1)	5,894,468 (15.3)	941 (14.2%)	964 (15.2%)	1124 (16.3%)	1133 (15.3%)	1115 (15.6%)	1117 (24.4%)	0.697
Mid-Low	6741 (24.4)	9,083,443 (23.6)	1246 (24.8%)	1241 (23.1%)	1427 (23.1%)	1394 (22.9%)	1433 (24.3%)	1510 (29.9%)	
Mid-High	7620 (27.6)	11,427,073 (29.7)	1427 (30.7%)	1382 (29.9%)	1590 (29.5%)	1588 (29.6%)	1633 (29.1%)	1500 (24.6%)	
High	7918 (28.6)	11,884,798 (30.9)	1351 (30.1%)	1465 (31.1%)	1651 (30.8%)	1685 (31.9%)	1766 (30.6%)	1769 (18.5%)	
Missing	86 (0.3)	151,565 (0.4)	16 (0.2%)	23 (0.7%)	15 (0.3%)	16 (0.4%)	16 (0.4%)	23 (2.57%)	
Educational Level									
<High school	8868 (32.1)	9,235,054 (24.0)	1730 (25.4%)	1734 (25.0%)	1846 (24.4%)	1813 (23.3%)	1745 (22.3%)	1642 (20.4%)	<0.001
High school graduate	8957 (32.4)	13,888,702 (36.1)	1642 (38.3%)	1705 (37.4%)	1843 (35.9%)	1777 (32.9%)	1990 (36.4%)	1978 (35.7%)	
≥College	9736 (35.2)	15,223,149 (39.6)	1588 (35.9%)	1623 (37.5%)	2099 (39.4%)	2210 (43.6%)	2216 (41.1%)	2292 (43.8%)	
Missing	81 (0.3)	94,442 (0.3)	21 (0.4%)	13 (0.2%)	19 (0.3%)	16 (0.2%)	12 (0.2%)	7 (0.1%)	
Smoking									
Non-smoker	16,680 (60.3)	21,850,769 (56.8)	3030 (56.9%)	3053 (56.8%)	3510 (56.6%)	3520 (57.4%)	3567 (56.6%)	3524 (56.7%)	<0.001
Past-smoker	5804 (21.0)	8,048,414 (20.9)	939 (18.6%)	1098 (21.4%)	1211 (21.0%)	1254 (21.5%)	1302 (22.0%)	1362 (23.0%)	
Current-smoker	4927 (17.8)	8,237,991 (21.4)	930 (22.9%)	840 (20.3%)	1057 (22.1%)	1029 (20.9%)	1071 (21.0%)	1020 (20.1%)	
Missing	231 (0.8)	304,173 (0.8)	82 (1.5%)	84 (1.5%)	29 (0.4%)	13 (0.2%)	23 (0.4%)	13 (0.2%)	
Alcohol drinking									
Non-drinking	3188 (11.5)	3,485,149 (9.1)	628 (9.7%)	607 (9.3%)	690 (9.3%)	658 (9.1%)	605 (8.0%)	626 (8.4%)	<0.001
Moderate-drinking	21,236 (76.8)	29,722,972 (77.3)	3772 (76.2%)	3902 (77.5%)	4456 (77.3%)	4476 (77.3%)	4630 (78.2%)	4626 (79.7%)	
Heavy-drinking	3047 (11.0)	5023,857 (13.1)	520 (12.9%)	497 (12.1%)	637 (13.1%)	673 (13.5%)	720 (13.8%)	656 (11.8%)	
Missing	171 (0.6)	209,369 (0.5)	61 (1.1%)	69 (1.2%)	24 (0.3%)	9 (0.2%)	8 (0.1%)	11 (0.2%)	

*N*, number.

**Table 2:** Changes in adherence rate to the aerobic PA guideline by survey year.

	The Adherence Rate to the Aerobic PA by Survey Year							
	2014	2015	2016	2017	2018	2019		
	% (95% CI)	% (95% CI)	% (95% CI)	% (95% CI)	% (95% CI)	% (95% CI)	*p* for Trend	Interaction
Total	57.0 (55.0–59.0)	51.0 (48.9–53.0)	47.7 (45.6–49.8)	46.4 (44.4–48.4)	44.9 (42.9–46.9)	45.6 (43.8–47.5)	<0.001	
Age (year)								
19–29	72.3 (68.1–76.0)	66.7 (62.3–70.9)	61.6 (57.0–66.0)	65.6 (61.5–69.4)	63.8 (58.9–68.5)	62.1 (57.8–66.1)	0.002	0.075
30–39	58.0 (54.1–61.8)	51.3 (46.6–56.1)	49.1 (45.5–52.8)	47.5 (43.3–51.8)	50.7 (46.5–55.0)	49.2 (45.1–53.4)	0.003	
40–49	57.2 (53.4–60.9)	54.5 (50.6–58.4)	47.9 (44.2–51.6)	47.5 (43.7–51.3)	45.8 (42.4–49.4)	45.3 (42.2–48.4)	<0.001	
50–59	56.3 (52.6–60.0)	46.3 (42.4–50.2)	45.4 (41.7–49.2)	44.4 (41.0–47.9)	37.9 (34.5–41.4)	41.4 (38.0–44.9)	<0.001	
60–69	47.8 (43.6–52.0)	45.7 (41.8–49.7)	45.7 (42.1–49.4)	35.8 (32.0–39.7)	38.6 (34.9–42.5)	39.3 (35.9–42.9)	<0.001	
70 or above	36.9 (33.3–40.7)	29.7 (25.9–33.7)	28.5 (25.3–32.0)	27.1 (23.7–30.7)	23.4 (20.3–26.7)	30.3 (26.9–34.1)	0.001	
Sex								
Male	61.5 (58.9–64.1)	54.6 (51.8–57.4)	51.2 (48.4–53.9)	49.0 (46.4–51.7)	48.7 (46.2–51.2)	50.3 (47.9–52.8)	<0.001	0.936
Female	52.7 (50.0–55.4)	47.5 (44.9–50.1)	44.3 (41.9–46.8)	43.8 (41.3–46.2)	41.1 (38.8–43.4)	41.0 (38.7–43.4)	<0.001	
Body Mass Index								
Underweight	50.3 (42.7–58.0)	45.6 (37.9–53.5)	39.9 (32.5–47.8)	46.1 (38.6–53.7)	46.8 (37.8–55.9)	40.7 (33.4–47.7)	0.176	0.37
Normal	58.0 (55.8–60.1)	51.7 (49.3–54.2)	48.6 (46.3–51.0)	46.4 (44.2–48.7)	45.2 (42.6–47.7)	46.7 (44.7–48.7)	<0.001	
Overweight	56.4 (52.8–59.9)	51.0 (47.4–54.7)	47.4 (44.1–50.7)	45.8 (42.9–48.8)	44.5 (41.6–47.4)	44.7 (41.3–48.1)	<0.001	
Obesity	54.4 (47.2–61.5)	46.3 (39.2–53.5)	45.1 (38.3–52.1)	49.3 (43.0–55.5)	42.7 (36.7–48.9)	43.4 (37.4–49.7)	0.023	
Family Income								
Low	47.0 (42.7–51.3)	41.1 (36.6–45.7)	36.8 (32.1–41.7)	33.9 (30.3–37.8)	36.4 (32.0–41.0)	35.2 (31.2–39.5)	<0.001	0.681
Mid-Low	56.4 (52.5–60.2)	50.0 (46.3–53.7)	44.2 (41.2–47.1)	47.3 (44.0–50.6)	41.4 (38.0–44.8)	44.1 (41.1–47.1)	<0.001	
Mid-High	58.3 (55.2–61.3)	52.7 (48.7–56.8)	48.6 (45.7–51.6)	46.1 (42.9–49.3)	46.0 (42.7–49.4)	47.2 (43.8–50.7)	<0.001	
High	61.2 (58.0–64.2)	54.8 (51.5–58.0)	55.6 (52.3–58.8)	52.0 (48.8–55.2)	51.0 (47.7–54.3)	50.2 (47.4–53.1)	<0.001	
Educational Level								
<High school	42.3 (39.2–45.4)	36.9 (33.7–40.2)	32.9 (30.2–35.6)	30.0 (27.4–32.8)	27.0 (24.4–29.9)	31.1 (28.1–34.3)	<0.001	0.394
High school graduate	62.7 (59.9–65.4)	55.7 (52.6–58.8)	51.7 (48.5–54.8)	50.7 (47.5–54.0)	48.7 (45.6–51.7)	48.8 (46.1–51.5)	<0.001	
≥College	61.4 (58.4–64.3)	55.7 (52.6–58.7)	53.6 (50.8–56.3)	52.0 (49.2–54.7)	51.3 (48.5–54.1)	49.9 (47.2–52.6)	<0.001	
Smoking								
Non-smoker	56.3 (53.6–59.0)	50.3 (47.5–53.0)	48.2 (45.6–50.7)	45.8 (43.4–48.2)	44.7 (42.2–47.1)	43.7 (41.4–46.0)	<0.001	0.352
Past-smoker	61.1 (57.3–64.8)	54.1 (50.3–58.0)	48.0 (44.4–51.6)	47.6 (44.3–50.9)	46.0 (42.4–49.7)	48.3 (44.8–51.7)	<0.001	
Current-smoker	56.7 (52.9–60.4)	50.5 (46.3–54.6)	46.7 (42.8–50.5)	46.7 (42.8–50.7)	44.1 (40.2–48.1)	48.2 (44.3–52.1)	<0.001	
Alcohol drinking								
Non-drinking	47.6 (42.2–53.1)	41.3 (36.4–46.2)	37.6 (33.3–42.1)	32.5 (28.0–37.2)	30.3 (25.9–35.1)	35.6 (31.1–40.3)	<0.001	0.095
Moderate-drinking	58.6 (56.4–60.8)	52.7 (50.3–55.0)	48.7 (46.5–51.0)	47.5 (45.3–49.7)	46.0 (43.8–48.3)	46.3 (44.3–48.2)	<0.001	
Heavy-drinking	56.3 (51.4–61.1)	49.1 (44.5–53.8)	49.8 (44.6–55.0)	49.5 (44.6–54.5)	46.6 (42.3–50.9)	48.5 (43.7–53.4)	0.015	

PA, physical activity. CI, confidence interval.

**Table 3:** Changes in adherence rate to the muscle-strengthening PA guideline by survey year.

	The Adherence Rate to the Muscle-Strengthening PA by Survey Year							
	2014	2015	2016	2017	2018	2019		
	% (95% CI)	% (95% CI)	% (95% CI)	% (95% CI)	% (95% CI)	% (95% CI)	*p* for Trend	Interaction
Total	20.8 (19.3, 22.3)	22.2 (20.7, 23.8)	20.7 (19.4, 22.0)	21.4 (20.1, 22.7)	22.3 (20.9, 23.7)	23.3 (21.9, 24.7)	0.976	
Age (year)								
19–29	28.6 (24.9, 32.5)	32.1 (28.1, 36.2)	30.2 (26.3, 34.4)	27.2 (23.8, 30.8)	31.5 (27.9, 35.3)	33.2 (29.2, 37.4)	0.292	0.149
30–39	17.7 (15.0, 20.8)	18.5 (15.6, 21.8)	14.6 (12.1, 17.5)	19.4 (16.7, 22.5)	21.4 (18.5, 24.7)	22.0 (18.7, 25.7)	0.03	
40–49	19.0 (16.2, 22.2)	22.1 (19.2, 25.3)	19.6 (17.2, 22.2)	19.5 (17.1, 22.1)	21.4 (18.6, 24.4)	20.2 (17.5, 23.2)	0.689	
50–59	21.8 (19.1, 24.7)	20.5 (17.5, 23.9)	22.4 (19.7, 25.4)	23.0 (20.1, 26.1)	20.0 (17.2, 23.1)	23.3 (20.6, 26.4)	0.603	
60–69	20.1 (16.8, 23.9)	22.8 (19.4, 26.6)	22.1 (18.8, 25.8)	22.4 (19.5, 25.6)	22.0 (19.0, 25.4)	22.5 (19.8, 25.6)	0.792	
70 or above	14.1 (11.5, 17.2)	14.0 (11.5, 17.0)	12.4 (10.3, 15.0)	14.5 (11.7, 17.7)	14.6 (12.3, 17.3)	15.7 (13.3, 18.5)	0.459	
Sex								
Male	29.2 (26.8, 31.6)	31.2 (28.6, 33.8)	27.0 (25.0, 29.0)	27.7 (25.7, 29.8)	30.5 (28.3, 32.6)	32.6 (30.3, 35.0)	0.416	0.953
Female	12.8 (11.4, 14.4)	13.8 (12.3, 15.5)	14.5 (13.0, 16.3)	15.1 (13.6, 16.7)	14.2 (12.7, 15.9)	14.1 (12.7, 15.6)	0.667	
Body Mass Index								
Underweight	16.7 (11.8, 23.0)	15.5 (10.4, 22.3)	12.3 (7.6, 19.2)	16.1 (11.2, 22.6)	23.6 (17.0, 31.8)	16.4 (12.1, 21.8)	0.404	0.255
Normal	21.6 (19.9, 23.4)	23.5 (21.7, 25.4)	21.5 (19.8, 23.3)	23.5 (21.9, 25.2)	23.3 (21.5, 25.2)	23.9 (22.2, 25.7)	0.366	
Overweight	20.0 (17.5, 22.8)	21.3 (18.5, 24.3)	21.5 (19.2, 24.0)	18.9 (16.9, 21.1)	21.6 (19.2, 24.2)	24.5 (22.1, 27.1)	0.368	
Obesity	17.9 (12.3, 25.1)	18.8 (13.1, 26.4)	13.0 (9.4, 17.7)	14.4 (10.4, 19.8)	14.3 (10.5, 19.3)	15.7 (11.4, 21.1)	0.266	
Family Income								
Low	12.6 (10.2, 15.4)	16.3 (13.3, 19.7)	17.1 (13.9, 20.9)	15.6 (12.7, 19.0)	13.4 (10.7, 16.7)	16.9 (14.1, 20.0)	0.489	0.136
Mid-Low	20.2 (17.4, 23.3)	23.2 (20.4, 26.1)	17.1 (14.8, 19.7)	20.5 (18.2, 23.1)	19.3 (16.9, 22.0)	21.4 (19.2, 23.7)	0.459	
Mid-High	22.4 (19.8, 25.3)	22.3 (19.6, 25.2)	21.0 (18.9, 23.3)	21.3 (19.0, 23.8)	22.5 (20.2, 25.0)	23.7 (21.2, 26.4)	0.934	
High	23.6 (21.3, 26.0)	24.0 (21.2, 27.1)	25.1 (22.6, 27.6)	24.8 (22.7, 27.0)	28.5 (26.0, 31.1)	27.1 (24.2, 30.2)	0.03	
Educational Level								
<High school	14.4 (12.4, 16.7)	13.3 (11.6, 15.3)	12.0 (10.4, 13.9)	14.1 (12.0, 16.4)	11.8 (9.9, 14.0)	14.7 (12.8, 16.7)	0.878	0.364
High school graduate	22.0 (19.4, 24.7)	26.3 (23.7, 29.1)	23.0 (20.8, 25.5)	22.9 (20.7, 25.4)	23.8 (21.6, 26.1)	24.8 (22.5, 27.2)	0.581	
≥College	24.2 (21.8, 26.8)	24.2 (21.5, 27.1)	24.0 (21.8, 26.3)	24.2 (22.3, 26.1)	26.6 (24.5, 28.9)	26.1 (23.8, 28.4)	0.221	
Smoking								
Non-smoker	17.8 (16.1, 19.6)	18.8 (17.1, 20.6)	18.3 (16.8, 20.0)	18.5 (17.0, 20.0)	19.3 (17.6, 21.1)	19.3 (17.7, 21.0)	0.542	0.719
Past-smoker	28.4 (25.0, 31.9)	30.6 (27.3, 34.2)	27.1 (24.2, 30.2)	30.0 (27.4, 32.9)	30.6 (27.6, 33.7)	30.2 (27.1, 33.6)	0.787	
Current-smoker	22.6 (19.7, 25.9)	23.0 (19.7, 26.6)	20.8 (17.9, 23.9)	20.4 (17.4, 23.8)	21.3 (18.5, 24.5)	26.5 (23.7, 29.6)	0.363	
Alcohol drinking								
Non-drinking	13.3 (10.3, 17.0)	11.9 (8.8, 15.8)	13.2 (10.2, 17.0)	13.7 (10.9, 17.0)	13.3 (9.7, 18.2)	11.7 (9.1, 15.1)	0.965	0.493
Moderate-drinking	21.3 (19.6, 23.0)	23.3 (21.5, 25.1)	21.3 (19.8, 22.9)	22.4 (20.9, 23.9)	23.1 (21.6, 24.8)	24.3 (22.6, 26.0)	0.14	
Heavy-drinking	24.5 (20.3, 29.1)	24.8 (20.3, 29.8)	22.6 (19.0, 26.7)	21.0 (17.7, 24.8)	22.7 (19.3, 26.4)	24.7 (20.9, 29.1)	0.485	

PA, physical activity. CI, confidence interval.

**Table 4:** Changes in adherence rate to both aerobic and muscle-strengthening PA guidelines by survey year.

	The Adherence Rate to Both Guidelines by Survey Year							
	2014	2015	2016	2017	2018	2019		
	% (95% CI)	% (95% CI)	% (95% CI)	% (95% CI)	% (95% CI)	% (95% CI)	*p* for Trend	Interaction
Total	15.6 (14.2, 17.0)	16.2 (14.8, 17.7)	14.4 (13.2, 15.6)	13.6 (12.5, 14.8)	14.3 (13.2, 15.5)	15.7 (14.5, 17.1)	0.051	
Age (year)								
19–29	25.1 (21.2, 29.4)	28.2 (24.4, 32.4)	24.5 (20.8, 28.6)	22.1 (18.8, 25.7)	25.8 (22.2, 29.8)	27.7 (23.9, 31.9)	0.893	0.047
30–39	13.5 (11.1, 16.4)	15.1 (12.3, 18.4)	10.6 (8.6, 13.1)	13.2 (10.7, 16.1)	14.7 (12.2, 17.5)	16.7 (13.7, 20.1)	0.376	
40–49	14.0 (11.5, 16.9)	16.1 (13.4, 19.2)	14.0 (12.0, 16.3)	13.5 (11.4, 16.0)	13.1 (10.9, 15.7)	13.0 (10.8, 15.6)	0.037	
50–59	15.8 (13.4, 18.6)	12.8 (10.4, 15.7)	14.5 (12.2, 17.2)	12.0 (10.1, 14.2)	11.1 (9.3, 13.1)	13.2 (11.0, 15.8)	0.003	
60–69	11.8 (9.2, 15.0)	13.2 (10.7, 16.2)	13.9 (11.3, 17.0)	11.0 (8.7, 13.7)	12.0 (9.9, 14.6)	13.2 (11.0, 15.8)	0.303	
70 or above	7.8 (5.9, 10.4)	6.6 (5.0, 8.7)	5.1 (3.8, 6.7)	6.6 (5.2, 8.5)	5.7 (4.3, 7.5)	7.8 (6.1, 9.9)	0.716	
Sex								
Male	22.0 (19.7, 24.4)	23.2 (20.8, 25.8)	19.0 (17.2, 20.9)	17.9 (16.2, 19.8)	19.7 (17.9, 21.7)	22.7 (20.6, 24.8)	0.148	0.873
Female	9.4 (8.2, 10.8)	9.6 (8.3, 11.0)	9.9 (8.5, 11.4)	9.4 (8.2, 10.7)	8.9 (7.8, 10.2)	8.9 (7.8, 10.3)	0.096	
Body Mass Index								
Underweight	14.5 (9.9, 20.7)	11.9 (7.6, 18.1)	7.9 (4.2, 14.6)	10.6 (7.1, 15.6)	17.1 (11.2, 25.1)	10.5 (6.9, 15.7)	0.787	0.828
Normal	16.3 (14.7, 18.1)	17.2 (15.5, 19.1)	14.6 (13.2, 16.1)	15.0 (13.7, 16.4)	14.4 (13.0, 15.9)	16.3 (14.7, 17.9)	0.083	
Overweight	14.6 (12.3, 17.3)	15.1 (12.5, 18.0)	15.6 (13.5, 17.9)	11.7 (9.9, 13.7)	14.7 (12.7, 17.1)	16.2 (14.2, 18.5)	0.468	
Obesity	10.9 (6.9, 16.8)	14.7 (10.0, 21.0)	10.3 (6.9, 14.9)	10.7 (7.1, 15.8)	9.8 (6.8, 14.0)	11.7 (7.9, 17.1)	0.356	
Family Income								
Low	8.0 (6.0, 10.6)	11.0 (8.5, 14.1)	9.9 (7.4, 13.1)	8.6 (6.3, 11.5)	8.2 (5.9, 11.2)	8.8 (6.6, 11.6)	0.324	0.041
Mid-Low	15.2 (12.6, 18.4)	17.3 (14.7, 20.3)	10.8 (9.0, 12.9)	12.5 (10.6, 14.6)	11.9 (10.0, 14.1)	13.5 (11.7, 15.4)	0.004	
Mid-High	16.9 (14.4, 19.7)	16.0 (13.3, 19.0)	15.2 (13.2, 17.4)	13.8 (12.0, 15.9)	14.1 (12.1, 16.3)	17.1 (14.7, 19.7)	0.335	
High	18.0 (15.7, 20.6)	17.9 (15.3, 20.9)	18.8 (16.7, 21.1)	16.6 (14.8, 18.6)	19.3 (17.2, 21.6)	19.5 (17.0, 22.3)	0.748	
Educational Level								
<High school	8.6 (7.0, 10.5)	6.4 (5.1, 8.1)	6.1 (5.0, 7.5)	6.0 (4.7, 7.7)	5.0 (4.0, 6.3)	6.4 (5.2, 7.9)	0.038	0.188
High school graduate	17.3 (15.0, 19.8)	19.8 (17.4, 22.6)	15.8 (13.8, 18.0)	15.9 (14.0, 18.0)	15.7 (13.9, 17.7)	16.9 (15.0, 19.0)	0.204	
≥College	18.7 (16.5, 21.2)	19.1 (16.7, 21.9)	18.3 (16.3, 20.4)	16.0 (14.3, 17.7)	18.1 (16.2, 20.1)	19.1 (17.0, 21.4)	0.513	
Smoking								
Non-smoker	13.5 (11.9, 15.2)	13.9 (12.3, 15.6)	13.0 (11.6, 14.6)	11.5 (10.3, 12.8)	12.9 (11.5, 14.5)	12.9 (11.5, 14.5)	0.092	0.601
Past-smoker	20.5 (17.6, 23.7)	21.5 (18.3, 25.1)	18.0 (15.5, 20.7)	18.7 (16.3, 21.4)	17.9 (15.2, 20.9)	19.4 (16.9, 22.2)	0.091	
Current-smoker	17.5 (14.8, 20.6)	17.2 (14.2, 20.7)	14.5 (12.1, 17.3)	14.1 (11.7, 17.0)	13.9 (11.7, 16.6)	19.4 (16.6, 22.5)	0.872	
Alcohol drinking								
Non-drinking	8.8 (6.1, 12.6)	7.3 (4.8, 11.0)	7.0 (4.8, 10.1)	6.2 (4.2, 9.0)	8.4 (5.2, 13.2)	6.4 (4.3, 9.4)	0.534	0.692
Moderate-drinking	16.3 (14.8, 18.0)	17.4 (15.8, 19.1)	14.9 (13.6, 16.4)	14.3 (13.1, 15.7)	14.7 (13.4, 16.0)	16.5 (15.0, 18.1)	0.059	
Heavy-drinking	17.0 (13.3, 21.5)	16.6 (12.9, 21.0)	16.6 (13.4, 20.5)	14.6 (11.8, 17.8)	15.7 (13.0, 18.8)	17.2 (14.2, 20.7)	0.635	

PA, physical activity. CI, confidence interval.

## Data Availability

Data used in this study are available on the website Korea National Health & Nutrition Examination Survey at “https://knhanes.kdca.go.kr/knhanes/sub03/sub03_02_05.do” (accessed on 1 November 2021).
